# Low prevalence of methicillin resistant *Staphylococcus aureus* as determined by an automated identification system in two private hospitals in Nairobi, Kenya: a cross sectional study

**DOI:** 10.1186/s12879-014-0669-y

**Published:** 2014-12-14

**Authors:** Geoffrey Omuse, Beatrice Kabera, Gunturu Revathi

**Affiliations:** Aga Khan University Hospital Nairobi, Nairobi, Kenya; Gertrudes Children’s Hospital, Nairobi, Kenya

**Keywords:** Staphylococcus aureus, Antibiotic susceptibility, MRSA

## Abstract

**Background:**

*Staphylococcus aureus* (*S.aureus*) is a major cause of both healthcare and community acquired infections. In developing countries, manual phenotypic tests are the mainstay for the identification of staphylococci with the tube and slide coagulase tests being relied upon as confirmatory tests for *S. aureus*. The subjectivity associated with interpretation of these tests may result in misidentification of coagulase negative staphylococci as *S.aureus*. Given that antibiotic resistance is more prevalent in CONS, this may result in over estimation of methicillin resistant *S.aureus* (MRSA) prevalence.

**Methods:**

A review of susceptibility data from all non-duplicate *S.aureus* isolates generated between March 2011 and May 2013 by the Vitek-2 (bioMérieux) automated system was performed by the authors. The data was generated routinely from processed clinical specimens submitted to the microbiology laboratories for culture and sensitivity at the Aga Khan University Hospital and Gertrude’s children’s hospital both situated in Nairobi.

**Results:**

Antimicrobial susceptibility data from a total of 731 non-duplicate *S.aureus* isolates was reviewed. Majority (79.2%) of the isolates were from pus swabs. Only 24 isolates were both cefoxitin and oxacillin resistant while 3 were resistant to oxacillin but susceptible to cefoxitin giving an overall MRSA prevalence of 3.7% (27/731). None of the isolates were resistant to mupirocin, linezolid, tigecycline, teicoplanin or vancomycin.

**Conclusion:**

The prevalence of MRSA in this study is much lower than what has been reported in most African countries. The significant change in antibiotic susceptibility compared to what has previously been reported in our hospital is most likely a consequence of the transition to an automated platform rather than a trend towards lower resistance rates.

**Electronic supplementary material:**

The online version of this article (doi:10.1186/s12879-014-0669-y) contains supplementary material, which is available to authorized users.

## Background

*Staphylococcus aureus* (*S. aureus*) is a major cause of both healthcare and community acquired infections and is perhaps the single most common cause of healthcare-associated infection throughout the world [1]-[3]. *S. aureus* has developed resistance to virtually all antibiotic classes available for clinical use and this has been observed in various continents [1],[3]-[7]. In Africa, methicillin resistant *S.aureus* (MRSA) prevalence is quite variable with prevalence’s as low as 4% and as high as 82% being reported [8].

A study carried out in Kenya in 1997 reported an MRSA prevalence of 39.8% amongst *S.aureus* isolates from a variety of clinical specimens at the national referral hospital in Nairobi [6]. A study looking at MRSA prevalence in 8 African countries found it to be between 20% and 30% in Nigeria, Cameroon and Kenya. The prevalence was less than 10% in Tunisia and Algeria. Only 137 isolates from Kenya were assessed in this study [4]. A retrospective analysis of bloodstream isolates at the Aga Khan University Hospital Nairobi (AKUHN) in Kenya reported a 21% prevalence of MRSA. This study looked at data obtained from 364 non duplicate isolates collected from January 2003 to April 2008 [9]. A 2013 publication looking at *S.aureus* isolates causing skin and soft tissue infections in 5 government run healthcare facilities in Nairobi reported MRSA prevalence amongst *S.aureus* to be 84.1%. This was out of 82 isolates collected between 2005 and 2007 [10]. In most of the mentioned studies, identification of *S.aureus* was performed using manual methods.

In developing countries, phenotypic tests are the mainstay for the identification of staphylococci with the tube and slide coagulase tests being relied upon as confirmatory tests for *S.aureus*. This is largely because human plasma is readily available in most hospital based laboratories. Kateete *et al*. reported specificities for human and sheep plasma tube coagulase tests of 11% and 8% respectively in identifying *S.aureus* when compared to a polymerase chain reaction (PCR) assay detecting the *nuc* gene which is specific for *S.aureus* [11]. Sperber *et al*. demonstrated that tube coagulase is only reliable when a firm clot which doesn’t move on tipping the tube is considered a positive reaction [12]. The subjectivity in interpreting the tube coagulase contributes considerably to its low specificity which may result in coagulase negative staphylococci (CONS) being misidentified as *S.aureus*. Given that antimicrobial resistance especially to methicillin is more common in CONS [13], this can lead to over estimation of MRSA prevalence as well as erroneous reporting of increased levels of resistance to other antibiotics.

Automated systems offer better species identification than manual methods as this is based on a larger panel of standardized biochemical tests. In addition, antimicrobial susceptibility is objectively assessed by automated determination of minimum inhibitory concentrations (MICs) [14],[15]. The use of automated identification systems is fairly recent in Kenya and this transition may result in a significant change in reported antimicrobial susceptibility patterns. The AKUHN started using an automated identification system for routine diagnosis in January 2011 while Gertrude’s Children’s Hospital (GCH) has used one since 2009.

We set out to describe the antimicrobial susceptibility patterns of over 700 *S.aureus* isolates from routine clinical specimens as determined byVitek-2 (version 4.01, bioMerieux, Marcy-l’Etoile, France). Vitek-2 is an automated identification and susceptibility testing system that enables rapid determination of MICs. Its improved performance over earlier rapid systems is due to the larger number of wells in each card, enhanced optics, and new algorithms based on kinetic analyses of growth data. It has an Advanced Expert System (AES) which provides standardized interpretive reading of these MICs.

To the best of our knowledge, this is the first study in East Africa that has reported *S.aureus* antibiotic susceptibility for over 700 isolates all identified using automated systems. Given the large number of isolates and presumably better species identification, this study gives a more accurate picture of *S.aureus* antimicrobial susceptibility in Nairobi over the past 2 years and provides a baseline against which data generated from similar automated systems in East Africa can be compared against.

## Methods

AKUHN is a 300 bed private university teaching hospital that was awarded international accreditation by the Joint Commission International in September 2013. The hospitals main laboratory is ISO15189:2007 accredited by the South African National Accreditation Service (SANAS) since 2010. GCH is a dedicated pediatric hospital in Nairobi with a bed capacity of 105. Both hospitals offer primary and tertiary care services with clientele largely comprising middle to high social economic status individuals residing in Nairobi and its environs. Both hospital laboratories receive samples from a network of satellite clinics located in and around Nairobi.

A review of all consecutive non-duplicate *S.aureus* susceptibility data generated between March 2011 and May 2013 by the Vitek-2 (bioMérieux) automated system was performed by the authors. The data was generated routinely from processed clinical specimens submitted to the microbiology laboratories for culture and sensitivity. The Vitek-2 card AST P580 was used for susceptibility testing and interpretation of the MICs was based on Clinical Laboratory Standard Institute guidelines [16]. The antibiotics tested and reported included penicillin, oxacillin, cefoxitin, gentamicin, amikacin, trimethoprim/sulfamethoxazole (TMP/SMX), levofloxacin, moxifloxacin, teicoplanin, vancomycin, linezolid, tetracycline, tigecycline, rifampicin, mupirocin, clindamycin, erythromycin and tobramycin. For oxacillin a cut off ≥4 ug/ml was considered resistant while for cefoxitin, a positive screen by Vitek-2 was considered resistant. As per the CLSI guidelines, a *S.aureus* isolate found to be resistant to either cefoxitin or oxacillin was reported as an MRSA.

The AKUHN and GCH ethics committees gave permission for the use of antimicrobial susceptibility data obtained from cultures done on patient samples.

### Statistics

Antibiotic susceptibility was expressed as a percentage of all *S.aureus* isolates. Comparison of antibiotic susceptibility and sample types between AKUHN and GCH isolates was done using chi-square or fishers exact tests where appropriate. All analysis was two tailed. A p-value less than 0.05 was considered statistically significant. Statistical analysis was performed using SPSS version 21.

## Results

Antimicrobial susceptibility data from a total of 731 non-duplicate *S.aureus* isolates was reviewed with AKUHN and GCH contributing 529 and 202 respectively. Pus swabs formed the bulk of the specimens comprising 79.2% with majority coming from patients with skin and soft tissue infections. The distribution of the clinical specimens was as shown in Table 1.

**Table 1:** **Table showing the proportion of different specimen types from which** ***Staphylococcus aureus*** **isolates were obtained at AKUHN and GCH**

Specimen	AKUHN	GCH	Total
	n (%)	n (%)	n (%)
Pus swabs	395 (74.7%)	184 (91.1%)	579 (79.2%)
Blood	40 (7.6%)	6 (3.0%)	46 (6.3%)
Urine	8 (1.5%)	6 (3.0%)	14 (1.9%)
Screening swabs	48 (9.1%)	2 (1.0%)	50 (6.8%)
Lower respiratory tract	17 (3.2%)	1 (0.5%)	18 (2.5%)
Miscellaneous^a^	21 (4.0%)	3 (1.5%)	24 (3.3%)
**Total**	**529** **(100.0%)**	**202** **(100.0%)**	**731** **(100.0%)**

^a^These consisted of ascitic fluid, knee aspirates, vaginal swabs and isolates where the source was not indicated.

Only 24 isolates were both cefoxitin and oxacillin resistant while 3 were resistant to oxacillin but susceptible to cefoxitin. Including these 3 isolates, the overall MRSA prevalence was 3.7% (27/731). The 3 isolates were from skin and soft tissue infections and were all susceptible to teicoplanin, tigecycline, vancomycin, linezolid and rifampicin. One of the isolates was only resistant to oxacillin and penicillin while the other 2 isolates in addition to being resistant to oxacillin and penicillin were also resistant to erythromycin and tetracycline with intermediate susceptibility to levofloxacin. One of the isolates was also resistant to trimethoprim/sulfamethoxazole, tobramycin and moxifloxacin while the other had inducible clindamycin resistance. The MRSA prevalence in blood stream isolates was 6.5% (3/46).

None of the *S.aureus* isolates was resistant to mupirocin, vancomycin, teicoplanin, tigecycline or linezolid. Resistance was highest to penicillin at 92.2% and trimethoprim-sulfamethoxazole at 42.1% as shown in Table 2.

**Table 2:** **Table showing antibiotic susceptibility of** ***Staphylococcus aureus*** **isolates**

Antibiotic	Susceptible	Intermediate	Resistant
	No. (%)	No. (%)	No. (%)
Penicillin	57 (7.8%)	0 (0.0%)	674 (92.2%)
Oxacillin	704 (96.3%)	0 (0.0%)	27 (3.7%)
Cefoxitin	707 (96.7%)	0 (0.0%)	24 (3.3%)
Erythromycin	645 (88.2%)	1 (0.1%)	85 (11.7%)
Clindamycin^a^	658 (90.0%)	0 (0.0%)	73 (10.0%)
Gentamicin	710 (97.1%)	7 (1.0%)	14 (1.9%)
Tobramycin	708 (96.8%)	5 (0.7%)	18 (2.5%)
Levofloxacin	687 (94.0%)	31 (4.2%)	13 (1.8%)
Moxifloxacin	724 (99.1%)	1 (0.1%)	6 (0.8%)
Linezolid	731 (100.0%)	0 (0.0%)	0 (0.0%)
Mupirocin	731 (100.0%)	0 (0.0%)	0 (0.0%)
Rifampicin	725 (99.2%)	3 (0.4%)	3 (0.4%)
TMP/SMX^b^	423 (57.9%)	0 (0.0%)	308 (42.1%)
Tetracycline	618 (84.5%)	0 (0.0%)	113 (15.5%)
Tigecycline	731 (100.0%)	0 (0.0%)	0 (0.0%)
Teicoplanin	731 (100.0%)	0 (0.0%)	0 (0.0%)
Vancomycin	731 (100.0%)	0 (0.0%)	0 (0.0%)

^a^59 isolates were susceptible to clindamycin based on MICs but were reported as resistant as they had inducible clindamycin resistance.

^b^TMP/SMX-Trimethoprim/Sulfamethoxazole.

Comparison of susceptibility between AKUHN and GCH isolates showed significantly less susceptibility in AKUHN isolates to oxacillin, levofloxacin and tobramycin. Generally, isolates from GCH were more susceptible to the antibiotics tested as shown in Table 3.

**Table 3:** **Comparison of** ***S.aureus*** **antibiotic susceptibility between AKUHN and GCH isolates**

Antibiotic	AKUHN (N = 529)	GCH (N = 202)	P-value
	n (%)	n (%)	
Penicillin	46 (8.7%)	11 (5.4%)	0.166
Oxacillin	504 (95.3%)	200 (99.0%)	0.015
Erythromycin	459 (86.8%)	186 (92.1%)	0.054
Clindamycin^a^	473 (89.4%)	185 (91.6%)	0.412
Gentamicin	510 (96.4%)	200 (99.0%)	0.080
Tobramycin	506 (95.7%)	202 (100.0%)	0.001
Levofloxacin	487 (92.1%)	200 (99.0%)	0.000
Moxifloxacin	522 (98.7%)	202 (100.0%)	0.199
Linezolid	529 (100.0%)	202 (100.0%)	1.000
Mupirocin	529 (100.0%)	202 (100.0%)	1.000
Rifampicin	524 (99.1%)	201 (99.5%)	1.000
TMP/SMX^b^	317 (59.9%)	106 (52.5%)	0.079
Tetracycline	446 (84.3%)	172 (85.1)	0.820
Tigecycline	529 (100.0%)	202 (100.0%)	1.000
Teicoplanin	529 (100.0%)	202 (100.0%)	1.000
Vancomycin	529 (100.0%)	202 (100.0%)	1.000

^a^After adjusting for inducible clindamycin resistance.

^b^TMP/SMX-Trimethoprim/Sulfamethoxazole.

**Figure 1:**
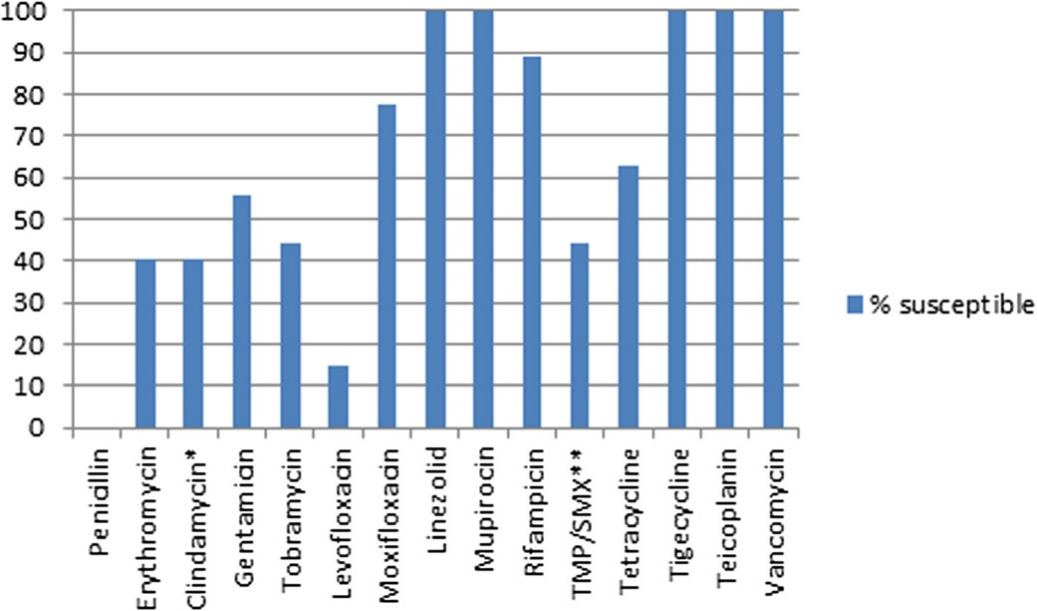
**Antibiotic susceptibility of MRSA isolates** **(N = 27)** **.** ^*^After adjusting for inducible clindamycin ;resistance. ^**^TMP/SMX-Trimethoprim/Sulfamethoxazole.

All MRSA isolates were susceptible to tigecycline, mupirocin, linezolid, teicoplanin and vancomycin as shown in Figure 1.

## Discussion

Antimicrobial susceptibility surveillance is important as it aids in identifying local resistance trends which impacts on the management of both hospital and community-acquired infections. We have previously reported an MRSA prevalence of 21% in *S.aureus* bacteremia isolates collected between January 2003 and April 2008 at AKUHN [9]. This was a retrospective review of laboratory susceptibility data that relied on the use of manual identification methods and susceptibility by disc diffusion. The overall MRSA prevalence of 3.7% for all specimen types and 6.5% in blood isolates in this study is therefore much lower than what was anticipated. Whether this reflects a true decline in methicillin resistance or is a result of better diagnostic methods is a question that can only be answered by continuous monitoring of trends in *S.aureus* susceptibility. A study carried out in 2010 that investigated nasal carriage of MRSA by healthcare workers (HCWs) at AKUHN found that 45 out of 246 randomly selected HCWs were carriers of *S.aureus* but none of the isolates were MRSA even after performing genotypic tests [17]. This low prevalence is in complete contrast to a recently published study that reported MRSA prevalence in *S.aureus* isolates from 5 public hospitals in Nairobi to be 84.1% [10]. In this study, manual bench techniques were used to identify *S.aureus* and to perform antimicrobial susceptibility. In as much as the patient population in public and private hospitals differ in terms of the social economic status, it is unlikely that this can explain the marked difference in MRSA prevalence. We hypothesize that the marked differences in MRSA prevalence amongst various hospitals in Nairobi is a consequence of the different laboratory techniques used to correctly identify MRSA.

A systematic review looking at MRSA in Africa found no decreasing trend in MRSA prevalence in individual countries except possibly for South Africa. This review included only articles published after 2005 and that had more than 100 isolates analyzed. Very few countries reported an MRSA prevalence less than 10% [8]. The low prevalence we report is however not unique in Africa. In Madagascar, Randrianirina *et al*. reported prevalence’s of 4.4% and 6.5% in hospital and community acquired *S.aureus* isolates respectively. Most of the isolates were community-acquired and largely originated from genital, urinary and pus specimens collected between January 2001 and December 2005 [18]. In Eritrea, a prevalence of 9% was reported in *S.aureus* isolates from pus and ear discharge [19]. In Gabon, the prevalence was 5.8% in isolates obtained from a variety of specimens collected between 2009 and 2012 [20]. In most of these studies, antimicrobial susceptibility testing was performed using disc diffusion and not an automated system.

Out of 27 oxacillin resistant isolates in this study, 3 were cefoxitin susceptible. A possible mechanism of resistance in these isolates is hyper-production of beta lactamase as is commonly found in Borderline oxacillin resistant *S.aureus* (BORSA) isolates [21]. However, these isolates all had oxacillin MICs >4 ug/ml. Typically, BORSA isolates have an MIC between 1 and 4 ug/mL. The clinical significance of this mechanism of resistance is not known but such isolates are still reported as MRSA due to the possibility of treatment failure if beta lactam antibiotics are used [22]. Confirmation of the mechanism of resistance in these isolates is required given the trend towards adoption of PCR based diagnostic technologies targeted at identifying only the *mecA* gene. In addition, some of the chromogenic plates used to identify MRSA are unable to identify those that do not have the *mecA* gene [23].

Resistance to trimethoprim/Sulfamethoxazole (TMP/SMX) was 42.1%. Various African studies have reported resistance ranging from 0% to 100% [8]. However, the marked heterogeneity in these studies makes it difficult to comment on the reasons for the differences seen. In South Africa, Kwa Zulu Natal province, 30.8% of *S.aureus* isolated in 2001 and 2002 from various clinical specimens were resistant to TMP/SMX. A study looking at blood isolates in 7 private pathology practices in South Africa reported resistance of 29% [24]. In Gabon, non-susceptible isolates only comprised 8.3% of all *S.aureus* isolates from non-invasive specimens [20]. A study in Ghana looking at 308 *S.aureus* isolates from diverse specimens found only 4% to be resistant to TMP/SMX [25]. TMP/SMX is a cheap oral drug with good bio-availability and broad spectrum cover that has been thought to be an ideal alternative in treating skin and soft tissue infections (SSIs) caused by MSSA and community acquired MRSA (CA-MRSA) [26],[27]. The high resistance seen in this study rules it out as an option for empiric treatment of SSIs at AKUHN and GCH.

Resistance to erythromycin and clindamycin was 11.7% and 10.0% respectively. Inducible clindamycin resistance was seen in 8.1% of isolates that were susceptible based on CLSI MIC cut offs [16]. Failure to test for inducible clindamycin resistance would have resulted in clindamycin resistance being reported as 1.9%. This highlights the importance of modifying clindamycin susceptibility for inducible phenotypes given that failure to do so can result in patients being treated with clindamycin which could result in treatment failure [28].

All isolates were susceptible to mupirocin, linezolid, vancomycin, teicoplanin and tigecycline. Generally, *S.aureus* resistance to most of these antibiotics is low in Africa [8]. Rifampicin resistance was 11.1% in MRSA isolates compared to 52.8% in MRSA isolates from public diagnostic laboratories in South Africa [29]. Given South Africa’s high incidence of tuberculosis and subsequent widespread use of rifampicin, it has been hypothesized that selective pressure has resulted in the emergence of rifampicin resistant MRSA.

Isolates from AKUHN were generally more resistant to most antibiotics compared to GCH isolates. We can only hypothesize that this may be a result of differences in antibiotic pressure in the two hospitals or may be as a result of the difference in population given that GCH only caters for the pediatric age group while AKUHN caters for mainly an adult population. The differences seen could also be a chance finding resulting from the multiple comparisons performed.

This study was limited by the fact that antibiotic resistance data was not stratified according to whether the infections were hospital or community acquired. Generally, community acquired isolates are less resistant compared to nosocomial isolates [30]. A second limitation is that the data was obtained only from 2 private hospitals in Nairobi hence limiting the generalizability of the results to other hospitals in Nairobi. Despite this limitation, AKUHN and GCH are both primary and tertiary healthcare facilities with a large network of satellite clinics and laboratories in and around Nairobi. Therefore, the susceptibility data presented from over 700 unique isolates can serve as a point of reference for the antimicrobial susceptibility of *S.aureus* isolates in Nairobi.

## Conclusions

The prevalence of MRSA in this study is much lower than what has been reported in most African countries. The significant change in antibiotic susceptibility compared to what has previously been reported is most likely a consequence of the transition to an automated platform rather than a trend towards lower resistance rates. It is likely that as more hospitals in Africa adopt similar systems, changes in previously reported susceptibility patterns will be observed.

## Authors’ original submitted files for images

Below are the links to the authors’ original submitted files for images. Authors’ original file for figure 1

## References

[1] Fluit AC, Wielders CL, Verhoef J, Schmitz FJ (2001). Epidemiology and susceptibility of 3,051 Staphylococcus aureus isolates from 25 university hospitals participating in the European SENTRY study. J Clin Microbiol.

[2] Lowy FD (1998). Staphylococcus aureus infections. N Engl J Med.

[3] McDonald LC (2006). Trends in antimicrobial resistance in health care-associated pathogens and effect on treatment. Clin Infect Dis.

[4] Kesah C, Ben Redjeb S, Odugbemi TO, Boye CS, Dosso M, Ndinya Achola JO, Koulla-Shiro S, Benbachir M, Rahal K, Borg M (2003). Prevalence of methicillin-resistant Staphylococcus aureus in eight African hospitals and Malta. Clin Microbiol Infect.

[5] Noskin GA, Rubin RJ, Schentag JJ, Kluytmans J, Hedblom EC, Smulders M, Lapetina E, Gemmen E (2005). **The burden of Staphylococcus aureus infections on hospitals in the United States**: **an analysis of the 2000 and 2001 Nationwide Inpatient Sample Database**. Arch Intern Med.

[6] Omari MA, Malonza IM, Bwayo JJ, Mutere AN, Murage EM, Mwatha AK, Ndinya-Achola JO (1997). Pattern of bacterial infections and antimicrobial susceptibility at the Kenyatta National Hospital, Nairobi, Kenya. East Afr Med J.

[7] Tiemersma EW, Bronzwaer SL, Lyytikainen O, Degener JE, Schrijnemakers P, Bruinsma N, Monen J, Witte W, Grundman H (2004). Methicillin-resistant Staphylococcus aureus in Europe, 1999–2002. Emerg Infect Dis.

[8] Falagas ME, Karageorgopoulos DE, Leptidis J, Korbila IP (2013). MRSA in Africa: filling the global map of antimicrobial resistance. PLoS One.

[9] Kohli R, Omuse G, Revathi G (2010). Antibacterial susceptibility patterns of blood stream isolates in patients investigated at the Aga Khan University Hospital Nairobi. East Afr Med J.

[10] Maina EK, Kiiyukia C, Wamae CN, Waiyaki PG, Kariuki S (2012). Characterization of methicillin-resistant Staphylococcus aureus from skin and soft tissue infections in patients in Nairobi, Kenya. Int J Infect Dis.

[11] Kateete DP, Kimani CN, Katabazi FA, Okeng A, Okee MS, Nanteza A, Joloba ML, Najjuka FC (2010). Identification of Staphylococcus aureus: DNase and Mannitol salt agar improve the efficiency of the tube coagulase test. Ann Clin Microbiol Antimicrob.

[12] Sperber WH, Tatini SR (1975). Interpretation of the tube coagulase test for identification of Staphylococcus aureus. Appl Microbiol.

[13] Diekema DJ, Pfaller MA, Schmitz FJ, Smayevsky J, Bell J, Jones RN, Beach M (2001). Survey of infections due to Staphylococcus species: frequency of occurrence and antimicrobial susceptibility of isolates collected in the United States, Canada, Latin America, Europe, and the Western Pacific region for the SENTRY Antimicrobial Surveillanc. Clin Infect Dis.

[14] Winstanley T, Courvalin P (2011). Expert systems in clinical microbiology. Clin Microbiol Rev.

[15] Spanu T, Sanguinetti M, Ciccaglione D, D’Inzeo T, Romano L, Leone F, Fadda G (2003). Use of the VITEK 2 system for rapid identification of clinical isolates of Staphylococci from bloodstream infections. J Clin Microbiol.

[16] CLSI (2010). Performance standards for antimicrobial susceptibility testing; Twentieth informational supplement. M100-S20, Vol. 29 No 3.

[17] Omuse G, Kariuki S, Revathi G (2012). Unexpected absence of meticillin-resistant Staphylococcus aureus nasal carriage by healthcare workers in a tertiary hospital in Kenya. J Hosp Infect.

[18] Randrianirina F, Soares J-L, Ratsima E, Carod J-F, Combe P, Grosjean P, Richard V, Talarmin A (2007). In vitro activities of 18 antimicrobial agents against Staphylococcus aureus isolates from the Institut Pasteur of Madagascar. Ann Clin Microbiol Antimicrob.

[19] Naik D, Teclu A (2009). A study on antimicrobial susceptibility pattern in clinical isolates of Staphylococcus aureus in Eritrea. Pan Afr Med J.

[20] Alabi AS, Frielinghaus L, Kaba H, Kösters K, Huson MA, Kahl BC, Peters G, Grobusch MP, Issifou S, Kremsner PG, Schaumburg F (2013). Retrospective analysis of antimicrobial resistance and bacterial spectrum of infection in Gabon, Central Africa. BMC Infect Dis.

[21] Chambers HF (1997). Methicillin resistance in staphylococci: molecular and biochemical basis and clinical implications. Clin Microbiol Rev.

[22] Skinner S, Murray M, Walus T, Karlowsky JA (2009). Failure of cloxacillin in treatment of a patient with borderline oxacillin-resistant Staphylococcus aureus endocarditis. J Clin Microbiol.

[23] Buchan BW, Ledeboer NA (2010). Identification of two borderline oxacillin-resistant strains of Staphylococcus aureus from routine nares swab specimens by one of three chromogenic agars evaluated for the detection of MRSA. Am J Clin Pathol.

[24] Brink A, Moolman J, da Silva MC, Botha M (2007). Antimicrobial susceptibility profile of selected bacteraemic pathogens from private institutions in South Africa. S Afr Med J.

[25] Egyir B, Guardabassi L, Sørum M, Nielsen SS, Kolekang A, Frimpong E, Addo KK, Newman MJ, Larsen AR (2014). Molecular Epidemiology and Antimicrobial Susceptibility of Clinical Staphylococcus aureus from Healthcare Institutions in Ghana. PLoS One.

[26] Roberts S, Chambers S (2005). Diagnosis and management of Staphylococcus aureus infections of the skin and soft tissue. Intern Med J.

[27] Sabol KE, Echevarria KL, Lewis JS (2006). Community-associated methicillin-resistant Staphylococcus aureus: new bug, old drugs. Ann Pharmacother.

[28] Drinkovic D (2001). Clindamycin treatment of Staphylococcus aureus expressing inducible clindamycin resistance. J Antimicrob Chemother.

[29] Marais E, Aithma N, Perovic O, Oosthuysen WF, Musenge E, Dusé AG (2009). Antimicrobial susceptibility of methicillin-resistant Staphylococcus aureus isolates from South Africa. S Afr Med J.

[30] Salmenlinna S, Lyytikäinen O, Vuopio-Varkila J (2002). Community-acquired methicillin-resistant Staphylococcus aureus, Finland. Emerg Infect Dis.

