# Supplementary figures and images for: Low prevalence of methicillin resistant Staphylococcus aureus as determined by an automated identification system in two private hospitals in Nairobi, Kenya: a cross sectional study
Source: BMC Infect Dis. 2014 Dec 14;14:669. doi: 10.1186/s12879-014-0669-y (PMC4269929; doi:10.1186/s12879-014-0669-y)

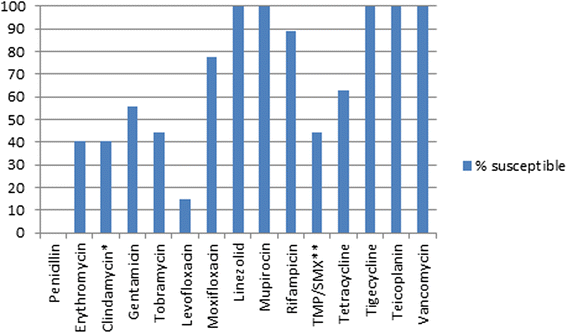

Supplement: Supplementary file 1 — Authors’ original file for figure 1 [file 12879_2014_669_MOESM1_ESM.gif]
